# Hepatitis B vaccination coverage and associated factors among medical students: a cross-sectional study in Bosaso, Somalia, 2021

**DOI:** 10.1186/s12889-023-15992-2

**Published:** 2023-06-03

**Authors:** Abdifitah Said Ali, Nur Ahmed Hussein, Elmi Omar Haji Elmi, Abdiwahid Mohamed Ismail, Mohamed Mohamud Abdi

**Affiliations:** 1 Faculty of Health Sciences, Global Science University (GSU), Galkayo, Somalia; 2 Faculty of Public Health, University of Health Sciences, Bosaso, Somalia; 3 Public Health Department, East Africa University, Bosaso, Somalia; 4 Mudug Regional Hospital, Galkayo, Somalia; 5 Puntland State Ministry of Health, Bosaso, Somalia

**Keywords:** Vaccination status, HBV, HCWs, Exposure

## Abstract

**Background:**

Hepatitis B virus (HBV) is a leading cause of liver cancer and remains a global public health concern. The risk of acquiring HBV is higher in HCWs than in non-HCWs. Medical students are considered a high-risk group because similar to HCWs, they tend to be exposed to body fluids and blood during training in clinical settings. New infections can be effectively prevented and eliminated with an increased coverage of HBV vaccination. The purpose of this study was to evaluate HBV immunization coverage and associated factors among medical students attending universities in Bosaso, Somalia.

**Methodology:**

An institutional-based cross-sectional study was conducted. A stratified sampling method was employed to draw a sample from four universities in Bosaso. From each university, participants were selected using a simple random sampling technique. Self-administered questionnaires were distributed among 247 medical students. The data were analysed with SPSS version 21, and the findings are presented in tables and proportions. The chi-square test was used to measure statistical associations.

**Results:**

Although 73.7% of the respondents had an above-average knowledge level of HBV and 95.9% knew that HBV can be prevented by vaccination, only 2.8% were fully immunized, while 5.3% were partially immunized. The students reported six main reasons for not being vaccinated: unavailability of the vaccine (32.8%), high vaccine cost (26.7%), fear of vaccine side effects (12.6%), lack of trust in vaccine quality (8.5%), lack of awareness about where to get vaccinated (5.7%), and lack of time (2.8%). Occupation and the availability of HBV vaccination in the workplace were associated with HBV vaccine uptake (*p* values of 0.005 and 0.047, respectively).

**Conclusion:**

HBV immunization coverage among medical students was extremely low (2.8%), indicating the urgent need for increased vaccination coverage in this population. This should start with evidence-based advocacy for the development of a clear national HBV elimination policy, followed by implementing effective, large-scale immunization strategies and interventions. Future studies should expand the sample size to include multiple cities for increased representativeness and conduct HBV titre tests among participants.

**Supplementary Information:**

The online version contains supplementary material available at 10.1186/s12889-023-15992-2.

## Background

Hepatitis B virus (HBV) remains a public health problem, with 257 million people (3.5% of the world’s population) living with chronic HBV infection as of 2015. The African region accounted for the highest prevalence rate (6.1%) in 2015 [1]. HBV infection is significantly more prevalent in African countries (> 8%) than in developed countries (< 2%) [2]. The prevalence of HBV among children under five years of age in Somalia was reported to be 11.06% as of 2019 [3].

HBV causes 80% of liver cancer cases and could be the second-most important human carcinogen after tobacco [4]. It caused 1.34 million deaths worldwide in 2015 alone, and mortality from HBV is on the rise, which is the opposite trend of human immunodeficiency virus (HIV) and tuberculosis [1].

HBV is 100 times more infectious than HIV [5]. In clinical settings, the virus can be transmitted through exposure to infected blood or other body fluids [6], as well as through percutaneous injury with contaminated instruments [2]. In addition, the virus can live outside the body for prolonged periods [7]. For instance, it can survive in dried blood for at least 7 days [8]. HBV can also be transmitted via discarded needles or fomites, even days after initial contamination [2].

Health care workers, including medical students, are considered a high-risk group due to occupational exposure [9]. Depending on location, 10–40% of health care workers have serologic evidence of HBV infection [10]. Furthermore, the risk of infection is 1%—6% when the patient is HBsAg-positive, and the transmission risk increases dramatically to 22%–40% if the patient is both HBsAg-positive and HBeAg-positive [2].

During clinical training, medical students may be more susceptible to occupational exposure, as they are relatively inexperienced [11], less aware than more experienced health care workers [2], and less likely to follow universal precautions while performing invasive procedures and handling high-risk fluids [12].

HBV vaccines have been available since 1982 and have been proven effective in preventing infection when administered before or immediately after exposure [13, 14]. Vaccinating new-borns within 24 h of birth is reported to be 90–95% effective in preventing HBV infection [13]. Thus, all health care workers, including clinical trainees (future HCWs), should receive an HBV vaccine as a routine occupational safety measure [2].

Unfortunately, HBV coverage is low among HCWs. The World Health Organization reported that a significant proportion of health care workers do not receive the HBV vaccine, with a coverage of 18–39% in low- and middle-income countries compared to 67–79% in high-income countries [9].

HBV is considered a substantial public health threat in Somalia, with a prevalence rate above 8% [3, 15]. HBV vaccination has been part of the national routine immunization program for children under five years of age in Somalia since 2013 [16]. There are no published data on HBV vaccination coverage among the general population or among medical students in the country. Therefore, this study aimed to evaluate HBV vaccination coverage and associated factors among medical students in Bosaso universities.

## Methodology

### Study design and study setting

An institution-based cross-sectional study was conducted at four universities in Bosaso city (East Africa University, University of Health Sciences, Red Sea University, and University of Bosaso) from March to June 2021. Bosaso is a coastal town located in the Gulf of Aden and is the capital of the northeastern region of Bari, Somalia.

### Study population

The study included all clinical students from the health sciences departments of the universities in Bosaso. These students were in the Medicine, Nursing, Midwifery, Clinical Officer, Medical Laboratory, and Dentistry Departments. The study participants were chosen from these departments because they have close contact with patients during their training period in hospitals, health centres, and clinics. Medical students who were not present at the time of the study, first year and second year students, and those who refused to participate were excluded.

### Sample size determination and sampling methods

To determine the number of study participants to be included in the study, the online Raosoft sample size calculator was used to calculate the sample size with a 5% margin of error and 95% CI. The target population was 683 clinical-level students from four different universities in Bosaso. The HBV vaccine coverage among clinical students was unknown; therefore, a maximum variation of 50% was assumed, and the final sample size was 247. The sample population was distributed among the four universities using a stratified sampling method. The research participants were selected from the universities using a simple random sampling technique.

### Data collection and analysis methods

The data were collected through self-administered questionnaires. The authors undertook the data collection process. The questionnaire was first prepared in English and then translated into the local language (Somali). The final draft questionnaire was pretested for face validity, and the two language versions were cross-checked for reconciliation before the actual data collection. The questionnaire contained four sections and 29 questions. Section A consisted of seven sociodemographic questions, Section B consisted of 11 knowledge questions, Section C consisted of seven HBV infection exposure history and laboratory testing questions, and Section D consisted of four HBV immunization status questions.

All participants responded. The data was entered into SPSS, where the variables were previously identified in English for data entry and analysis. Two of the researchers cross-checked the questionnaire data entered into SPSS against the collected paper questionnaires for completeness, consistency, and correctness before conducting the analysis. The data were analysed using SPSS version 21. Descriptive analysis was conducted, and frequencies and percentages of the variables were computed.

The questionnaire contained eleven questions with which we measured the level of HBV knowledge among the respondents. The selection of these questions and the overall questionnaire were adopted from the literature, and alignment with the study objectives was assured. To test the internal consistency of the knowledge measurement tool, Cronbach’s alpha was computed, which showed good internal consistency with a score of 0.85. The respondents were asked to answer the 11 questions with either 'Yes' or 'No'. Respondents who correctly answered 6 or more questions were considered to have above average knowledge of HBV, and those with fewer than 6 correct answers were considered to have below average knowledge of HBV. Finally, the proportion of respondents in the above average knowledge and below average knowledge categories was calculated.

A chi-square test was used to measure the association between HBV vaccination status as the dependent variable and sociodemographic status, knowledge of HBV, availability of HBV vaccine in the workplace and workplace HBV testing policy as independent variables. A *P* value of less than 0.05 considered statistically significant. The data quality was regularly checked throughout the study. The study results are presented in tables and proportions.

## Results

The sociodemographic characteristics of the participants are presented in Table 1. Of the 247 respondents, the majority were 25 years old (92.7%) or younger, and there were more females (71.3%) than males. A total of 10.9% were married. All of the participants attended medical training in clinical settings; in addition, 20% had part-time jobs in the medical field. The participants were studying in different departments: Clinical Laboratory (32.8%), Nursing (23.5%), Midwifery (19.8%), Medicine (10.1%), Clinical Officer (8.9%), and Dentistry (4.9%). More than half (53.4%) were in their fourth year of study. A total of 13.9% had a monthly income of more than $250.

**Table 1:** Sociodemographic characteristics

Age		
**Age group**	**Frequency**	**Percentage**
19–25	229	92.7%
26–30	16	6.5%
31 and older	2	0.8%
**Gender**		
**Gender**	**Frequency**	**Percentage**
Male	71	28.7%
Female	176	71.3%
**Occupational status**		
**Occupation**	**Frequency**	**Percentage**
Working in the medical field	50	20.0%
Working in a non-medical field	27	11.0%
Unemployed	170	69.0%
**University department of the respondents**		
**Department**	**Frequency**	**Percentage**
Medicine	25	10.1%
Clinical Laboratory	81	32.8%
Nursing	58	23.5%
Midwifery	49	19.8%
Clinical Officer	22	8.9%
Dentistry	12	4.9%
**Current year of study**		
**Year**	**Frequency**	**Percentage**
Third year	91	36.8%
Fourth year	132	53.4%
Fifth year	13	5.3%
Sixth year	11	4.5%
**Monthly income**		
**Income**	**Frequency**	**Percentage**
Less than $100	91	37.1%
$100–150	83	33.9%
$151–250	37	15.1%
> $250	34	13.9%
**Marital status**		
**Marital status**	**Frequency**	**Percentage**
Married	27	10.9%
Single	220	89.1%

Table 2 showsc the proportions of correct answers for the HBV knowledge questions among respondents. The percentage of correct answers for each knowledge question ranged between 58.8% and 95.9% for ‘whether HBV can be transmitted mother-to-child’ and ‘whether HBV can be prevented by immunization’, respectively.

**Table 2:** Knowledge of HBV among medical students in Bosaso Universities

	**Correct Answers**	
	N	Percent
**Mode of transmission**		
HBV can be transmitted through contact with the blood and body fluids of an infected person	215	87.8%
HBV can be transmitted through sexual contact	171	69.8%
HBV can be transmitted from mother to baby during delivery	144	58.8%
HBV can be transmitted through needle stick injury	163	66.5%
**Complications of HBV**		
HBV can cause chronic hepatitis	204	83.6%
HBV can cause liver cirrhosis	151	61.9%
HBV can cause hepatocellular carcinoma	155	63.5%
HBV can cause hepatic failure	169	69.3%
**Prevention Methods**		
HBV can be prevented through immunization	236	95.9%
HBV can be prevented by wearing appropriate personal protective equipment (PPE)	158	64.2%
HBV can be prevented by avoiding unsafe sex	155	63.0%

Overall, 73.7% of the respondents answered 6 or more questions correctly (above average knowledge).

According to the data in Table 3, approximately one-third of the respondents reported a history of accidental needle injury (30.4%) and accidental blood exposure (34.8%). There was an overall HBV testing rate of 37.2% among the participating students, but only 6.5% did so because of institutional requirements as a condition for either work or study. The majority (65.2%) were tested on a voluntary basis to determine their HBV status. Other testing reasons were blood donation (21.7%), giving birth (2.2%), and other (4.3%).

**Table 3:** Students’ exposure history at health facilities and HBV testing status

Accidental needle injury		*N* = 247
	Frequency	Percent
Yes	75	30.4%
Accidental blood exposure		*N* = 247
	Frequency	Percent
Yes	86	34.8%
HBV testing status		*N* = 247
	Frequency	Percent
Yes	92	37.2%
HBV testing reason		*N* = 92
	Frequency	Percent
I was donating blood	20	21.7%
I wanted to know my HBV status	60	65.2%
Institutional requirement	6	6.5%
I was giving birth	2	2.2%
Other reason	4	4.3%

Regarding the reason for not being vaccinated against HBV, more than half of the participants (59.5%) stated either 'unavailability of HBV vaccine' (32.8%) or 'high vaccine cost' (26.7%) as a barrier to receiving vaccination. Other responses were a fear of side effects (12.6%), a lack of trust in vaccine quality (8.5%), unaware of where to get vaccinated (5.7%), too busy (2.8%), unaware that an HBV vaccine exists (2%), not important (0.8%), and other (2.8%) (Table 4). A small proportion of the respondents reported that their universities (0.8%) and workplaces (10.7%) had HBV testing policies, and some (16.4%) reported that the HBV vaccine was available in these workplaces.

**Table 4:** HBV vaccination status among respondents and reasons for no or incomplete immunization

Received HBV vaccination	*N* = 247	
	Frequency	Percentage
Yes	13	5.3%
HBV vaccination fee		
	Frequency	Percentage
Self-paid	7	54%
Free	6	46%
Number of doses received		
	Frequency	Percentage
One dose	4	1.6%
Two doses	2	0.8%
Three doses	7	2.8%
Reason for not being vaccinated against HBV		
	Frequency	Percentage
The vaccine is not available	81	32.8%
High vaccine cost	66	26.7%
Afraid of vaccine side effects	31	12.6%
Do not trust vaccine quality	21	8.5%
I do not know where to go for vaccination	14	5.7%
Lack of time	7	2.8%
I'm not aware of an HBV vaccine	5	2.0%
Not important	2	0.8%
Other reason	7	2.8%
HBV testing policy at the university	*N* = 244	
	Frequency	Percentage
Yes	2	0.8%
HBV testing policy in the workplace	*N* = 75	
	Frequency	Percentage
Yes	8	10.7%
Availability of HBV vaccine at the workplace	*N* = 73	
	Frequency	Percentage
Yes	12	16.4%

The data in Table 5 indicate that the students' occupation and the availability of HBV vaccination in the workplace were associated with HBV vaccination status (X^2^ = 10.791 and 3.929, respectively; *P* value < 0.05). In contrast, age, monthly income, marital status, having a testing policy in the workplace, and HBV knowledge level were significantly associated with HBV immunization status.

**Table 5:** Factors associated with HBV vaccine uptake (chi-square test)

		HBV vaccine status		X^2^	*P* value
		Yes	No		
Age	21–25	11	218	1.889	0.389
	26–30	2	14		
	31 and older	0	2		
Occupational status	working in the medical field	7	43	10.791	0.005
	working in a non-medical field	2	25		
	unemployed	4	166		
Monthly income	Less than $100	1	90	5.66	0.129
	$100–150	7	76		
	$150–250	2	35		
	> $250	3	31		
Marital status	Married	3	24	2.079	0.149
	Unmarried	10	210		
HBV testing policy in the workplace	Available	2	6	2.047	0.153
	Not available	6	63		
HBV vaccination available in the workplace	Available	3	8	3.929	0.047
	Not available	5	61		
HBV knowledge level	Below average	8	174	1.044	0.307
	Above average	5	60		

## Discussion

HBV infection is considered a serious health problem in developing countries and can result in chronic liver cirrhosis, hepatocellular carcinoma, and hepatic failure [17]. Studies have indicated that the risk of acquiring HBV is four times higher in HCWs than in non-HCWs [18]. Clinical-level medical students are similarly considered to have a higher risk of HBV infection [19, 20]. This is because they can encounter occupational exposure to blood and body fluids during training in clinical settings [21, 22]. Low HBV vaccination coverage among medical students, a high rate of accidental exposure to blood and body fluids, and the relative inexperience of these students could increase the risk of viral transmission [11].

Despite the high HBV infection rates among medical students (up to 31.5%) in Africa [23], the effectiveness of HBV vaccination, and the WHO recommendation to immunize people at highest risk [24], only 2.8% of the respondents were fully vaccinated against HBV (Table 4). This rate is lower than those reported in Ethiopia (5.8%), Nigeria (34.8%), Cameron (18%), Kenya (20.2%), Uganda (44.3%), the Kingdom of Saudi Arabia (43.2%) and India [2, 25–30]. However, another previous study reported low HBV immunization coverage among medical university students [31].

Among the participants, barriers to immunization included HBV vaccine unavailability, a high vaccine cost, a fear of vaccine side effects, and a lack of trust in vaccine quality (Table 4). This is in line with several studies conducted in different countries that reported HBV vaccine unavailability, high cost, and a lack of training about infection prevention as reasons for no or incomplete immunization among medical students [26, 27, 30, 32].

The very low HBV vaccination coverage reported in this study could be partially attributed to the fact that all respondents were born in the pre-HBV vaccination era, as HBV immunization for children was introduced in Somalia for the first time in April 2013 as a component of a pentavalent vaccine (five-in-one vaccine) [16]. Another factor could be that few of the facilities and universities had an HBV testing policy. Notably, Somalia has no national HBV screening or vaccination policy for high-risk groups, including HCWs and medical students [33]. The introduction of opt-in and/or mandatory HBV vaccination in medical education institutions and workplaces would increase immunization coverage [34].

We found an above average level of HBV knowledge (73.6%). This finding was similar to those of other studies conducted in Ethiopia (73.6%) and Uganda (74.6%) [27, 30]. One study conducted in India found a lower HBV knowledge score (43%) [27]. This difference might be due to differences in the health policies of the countries.

Almost one-third of the participants had experienced unintentional needle injury during their training (Table 3), which is higher than that reported in Nigeria (almost half) and Syria (more than three quarters) [35, 36]. Approximately one-third of the respondents had at least one accidental exposure to blood during their medical training. This result is lower than that reported in Cameroon, where more than half of the participants had experienced accidental exposure to blood [25]. These differences might be due to the high levels of HBV knowledge among our study respondents.

Finally, the vaccination status of the respondents was significantly associated with their occupation and the availability of HBV vaccination in the workplace (Table 5). This is consistent with other studies that have shown an association between HBV vaccination status and marital status [37], income [38, 39], occupation [25, 39, 40], and an HBV vaccination policy at the workplace as a job entry requirement [41].

However, the vaccination status of the medical students was self-reported and not verified by vaccination records. Thus, there is a possibility of recall bias, leading to an over- or underestimation of immunization coverage.

## Conclusions

This study revealed a low vaccination coverage among medical students in Bosaso. Although the majority of respondents (73.3%) had above-average HBV knowledge scores, and 95.9% knew that HBV can be prevented by immunization, only 2.8% were fully vaccinated. From six reasons for not being vaccinated, 59.5% of the respondents reported HBV vaccine unavailability and the high cost of HBV vaccination as the main barriers to immunization. The chi-square test results showed that occupation and the availability of HBV vaccination in the workplace were associated with HBV vaccine uptake (*p* values of 0.005 and 0.047, respectively).

These findings will contribute to evidence-based communication about the HBV infection risk among Somali medical students and HCWs in general.

To raise HBV immunization coverage among these high-risk groups, advocacy for the development of a national HBV elimination policy should be accelerated. To strengthen the evidence base, future research should included a larger sample size that covers multiple cities for increased generalizability and conduct HBV titre testing.

## Supplementary Information

**Additional file 1.** Hepatitis B vaccination coverage and associated factors questionnaire.

## Data Availability

The data are available through the corresponding author upon reasonable request.

## Abbreviations

HBV: Hepatitis B virus
HIV: Human immunodeficiency syndrome
HBsAG: HBV surface antigen
HCWs: Health care workers
SPSS: Statistical Package for the Social Sciences
WHO: World Health Organization
