# Supplementary material for: Hepatitis B vaccination coverage and associated factors among medical students: a cross-sectional study in Bosaso, Somalia, 2021
Source: BMC Public Health. 2023 Jun 3;23:1060. doi: 10.1186/s12889-023-15992-2 (PMC10239102; doi:10.1186/s12889-023-15992-2)
Supplement: Supplementary file 1 — Additional file 1. Hepatitis B vaccination coverage and associated factors questionnaire. [file 12889_2023_15992_MOESM1_ESM.pdf]

Hepatitis B vaccination coverage and associated factors questionnaire

Data from this questionnaire will be used for research purposes only.

Researchers: Abdifitah Ali, Nur Hussein, Elmi Omar Haji Elmi, Abdiwahid Ismail, and Mohamed Abdi

Questionnaire No:…………..

Date of data collection (DD/MM/YY): (…………………)

CONSENT (Please read before filling out this questionnaire): By reading the research information contained in the participant information page, you are consenting to participate in this study by filling out this questionnaire.

Please complete the questionnaire only if you are 19 years or older and are in your third year of study or higher in one of these departments/faculties: Medicine, Nursing, Midwifery, Clinical Officer, Clinical Laboratory or Dentistry.

Note: Do not write your name on the questionnaire. Please answer all of the questions that pertain to you by following the instructions. Please answer truthfully.

Kindly check (✔) in the bracket of the option(s) that most correctly answers the question. Please make sure to choose only one option for each question unless the instruction advises otherwise.

Section A: Sociodemographic questions

1. Age
    a. 19 to 24 ( )
    b. 25 to 30 ( )
    c. 31 or older ( )
2. Sex
    a. Male ( )
    b. Female ( )
3. Occupation
    a. Working in the clinical field (e.g., nurse/lab technician/midwife/dental/clinical officer, etc.) ( )
    b. Working in non-medical field ( )
    c. Unemployed ( )
4. Faculty/Department
    a. Medicine ( )
    b. Clinical laboratory ( )
    c. Nursing ( )
    d. Midwifery ( )
    e. Clinical officer ( )
    f. Dentistry ( )
5. Current year of study
    a. Third year ( )
    b. Fourth year ( )

c. Fifth year ( )

d. Sixth year ( )

6. Monthly Income

a. Less than $100 ( )

b. $100-150 ( )

c. $151-250 ( )

d. >$250 ( )

7. Marital Status

a. Married ( )

b. Single ( )

SECTION B: KNOWLEDGE ABOUT HBV

1. HBV can be transmitted through contact with the blood and body fluids of an infected person.

a. Yes ( )

b. No ( )

2. HBV can be transmitted through sexual contact.

a. Yes ( )

b. No ( )

3. HBV can be transmitted from mother to baby during delivery.

a. Yes ( )

b. No ( )

4. HBV can be transmitted through contaminated needle stick injury.

a. Yes ( )

b. No ( )

5. HBV can cause chronic hepatitis.

   a. Yes ( )

   b. No ( )

6. HBV can cause liver cirrhosis.

   a. Yes ( )

   b. No ( )

7. HBV can cause hepatocellular carcinoma.

   a. Yes ( )

   b. No ( )

8. HBV can cause hepatic failure.

   a. Yes ( )

   b. No ( )

9. HBV can be prevented through immunization.

   a. Yes ( )

   b. No ( )

10. HBV can be prevented by wearing appropriate personal protective equipment (PPE).

    a. Yes ( )

    b. No ( )

11. HBV can be prevented by avoiding unsafe sex.

    a. Yes ( )

    b. No ( )

SECTION C: Exposure history at health facilities and HBV testing status

1. Have you ever had an accidental needle injury at a health facility?
   a. Yes ( )
   b. No ( )
2. Have you ever had an accidental blood exposure at a health facility?
   a. Yes ( )
   b. No ( )
3. Have you ever been tested for HBV?
   a. Yes ( )
   b. No ( )
4. If yes, what was the reason?
   a. Was donating blood ( )
   b. Wanted to know my HBV status ( )
   c. For an institutional obligation ( )
   d. For marriage reasons ( )
   e. I was giving birth ( )
   f. Other (please state)…………………………………………………
5. Does your university have an HBV testing policy?
   a. Yes ( )
   b. No ( )
6. Does your workplace have an HBV testing policy? Skip if you are unemployed.
   a. Yes ( )
   b. No ( )

7. Is HBV vaccination available in your workplace? Skip if you are unemployed.
    a. Yes ( )
    b. No ( )

SECTION D: HBV vaccination status and reasons for incomplete or no immunization.

1. Have you ever received the HBV vaccine? (if no skip to question 4)
    a. Yes ( )
    b. No ( )
2. If yes, the vaccination was
    a. Free ( )
    b. Self-paid ( )
3. If you have undergone HBV vaccination, how many doses have you received?
    a. One dose ( )
    b. Two doses ( )
    c. Three doses ( )
4. If you are not vaccinated against HBV, what is the reason(s)?
    a. High cost of the vaccine ( )
    b. Afraid of vaccine side effects ( )
    c. Do not trust vaccine quality ( )
    d. I do not know where to go to receive it ( )
    e. Lack of time ( )
    f. Not important ( )
    g. Other (please state)……………………………………………………………
